# Antibodies against human endogenous retrovirus K102 envelope activate neutrophils in systemic lupus erythematosus

**DOI:** 10.1084/jem.20191766

**Published:** 2021-05-21

**Authors:** Maria Tokuyama, Bronwyn M. Gunn, Arvind Venkataraman, Yong Kong, Insoo Kang, Tasfia Rakib, Michael J. Townsend, Karen H. Costenbader, Galit Alter, Akiko Iwasaki

**Affiliations:** 1 Department of Immunobiology, Yale University School of Medicine, New Haven, CT; 2 Ragon Institute of Massachusetts General Hospital, Massachusetts Institute of Technology, and Harvard University, Cambridge, MA; 3 Department of Internal Medicine, Yale University School of Medicine, New Haven, CT; 4 Biomarker Discovery OMNI, Genentech, South San Francisco, CA; 5 Department of Medicine, Brigham and Women’s Hospital, Boston, MA; 6 Howard Hughes Medical Institute, Chevy Chase, MD

## Abstract

Neutrophil activation and the formation of neutrophil extracellular traps (NETs) are hallmarks of innate immune activation in systemic lupus erythematosus (SLE). Here we report that the expression of an endogenous retrovirus (ERV) locus ERV-K102, encoding an envelope protein, was significantly elevated in SLE patient blood and correlated with autoantibody levels and higher interferon status. Induction of ERV-K102 in SLE negatively correlated with the expression of epigenetic silencing factors. Anti-ERV-K102 IgG levels in SLE plasma correlated with higher interferon stimulated gene expression, and further promoted enhanced neutrophil phagocytosis of ERV-K102 envelope protein through immune complex formation. Finally, phagocytosis of ERV-K102 immune complexes resulted in the formation of NETs consisting of DNA, neutrophil elastase, and citrullinated histone H3. Together, we identified an immunostimulatory ERV-K envelope protein that in an immune complex with SLE IgG is capable of activating neutrophils.

## Teaser

Using ERVmap, the authors determined that ERV-K102 expression was elevated in SLE patients’ peripheral blood cells and correlated with the interferon signature. IgG antibodies from SLE patients complexed with the K102 envelope protein activate neutrophils and induce NETs.

## Introduction

Systemic lupus erythematosus (SLE) is a complex and variable autoimmune disease that affects predominantly women of childbearing age. Hallmarks of disease include autoreactive T and B cells, immune complex deposition in tissues, and systemic activation of type I IFN signaling and cytokines (Tsokos et al., 2016). Billions of dollars have been spent on research and development and clinical trials over the past few decades, yet belimumab (monoclonal antibody against B-cell-activating factor of the tumour-necrosis-factor family, BAFF) is the only US Food and Drug Administration–approved targeted biological therapy for SLE (Navarra et al., 2011; Furie et al., 2011), and there is a great need to develop new effective therapies (Merrill et al., 2018).

Endogenous retroviruses (ERVs) are retroviral sequences that originated from exogenous retroviruses that integrated into our ancestral genome 2 to 40 million years ago and have persisted through generations (Stoye, 2012). ERV sequences make up as much as 8% of the human genome, in contrast to the 2% that encodes proteins (Lander et al., 2001). Exogenous retroviral genomes originally integrated as proviral sequences, similar to HIV, but most of the now endogenous sequences have acquired mutations over the course of evolution and rendered them replication incompetent (Stoye, 2012). In fact, roughly 90% of the ERV sequences that amount to hundreds of thousands of copies in the genome are solo LTRs resulting from homologous recombination between the 5′ and 3′ LTRs. A minority of ERVs represented by a few thousand copies have a relatively intact proviral structure, composed of some or all of the original open reading frames (Tristem, 2000; Subramanian et al., 2011; Schmitt et al., 2013a, 2013b; Vargiu et al., 2016).

Solo LTRs carry out important gene regulatory functions as alternative promoters and enhancers. They are proposed to have contributed to species evolution through the regulation of host gene networks and critical host genes, most notably those involved in embryogenesis and stem cell development (Feschotte, 2008; Jern and Coffin, 2008; Schlesinger and Goff, 2015; Chuong et al., 2017; Fuentes et al., 2018). Proviral ERVs have gained growing interest due to their association with diseases such as cancer and neurodegenerative diseases, with particular emphasis on the ERV-K family of ERVs, also known as HML-2 (Subramanian et al., 2011; Schmitt et al., 2013b; Garcia-Montojo et al., 2018). ERV-Ks are the only ERVs that are human specific with intact open reading frames, two of which remain unfixed in the human population (K113 and K115; Jha et al., 2011; Wildschutte et al., 2016; Li et al., 2019). In addition, ERV-Ks are the only ERVs reported to generate viral-like proteins in teratocarcinoma cell line and human blastocysts (Löwer et al., 1993; Bhardwaj et al., 2015; Grow et al., 2015).

ERV expression is largely suppressed epigenetically in somatic cells, but aberrant expression of ERVs has been implicated in disease, including SLE pathogenesis. Viral antigen related to the primate p30 gag protein is present at sites of active lupus glomerulonephritis (Mellors and Mellors, 1976). Antibody reactivity against whole virions or gag and env peptides from murine leukemia virus and baboon ERV (Blomberg et al., 1994) and ERV-derived ERV-9 and HRES-1 peptides (Bengtsson et al., 1996) are also observed in SLE. Roughly half of the SLE patients have reactivity against a 28-kD nuclear autoantigen (p28) that is encoded by a human T cell lymphotropic virus–related endogenous sequence (HRES-1; Banki et al., 1992; Perl et al., 1995; Perl et al., 2008). Several haplotypes of HRES-1 contained in the fragile site of chromosome 1 (1q42) are associated with disease (Pullmann et al., 2008). These studies have emphasized a strong association between ERVs and SLE, but there is little mechanistic understanding of how ERVs contribute to systemic inflammation in SLE. Furthermore, the potential roles of proviral ERV sequences, including ERV-K members in SLE, have not yet been reported.

We recently developed a tool called ERVmap to perform locus-specific proviral ERV transcriptome analysis of RNA sequencing (RNA-seq) data and revealed over 100 unique ERV loci that are significantly elevated in lupus peripheral blood mononuclear cells (PBMCs; Tokuyama et al., 2018). Here, we used ERVmap to further analyze an independent cohort of lupus patients to determine the role of proviral ERVs in systemic inflammation and potential mechanisms by which antibodies against ERVs may contribute to inflammation in SLE.

## Results

### Human-specific envelope-coding ERV-K loci are elevated in lupus blood

**Figure 1.**
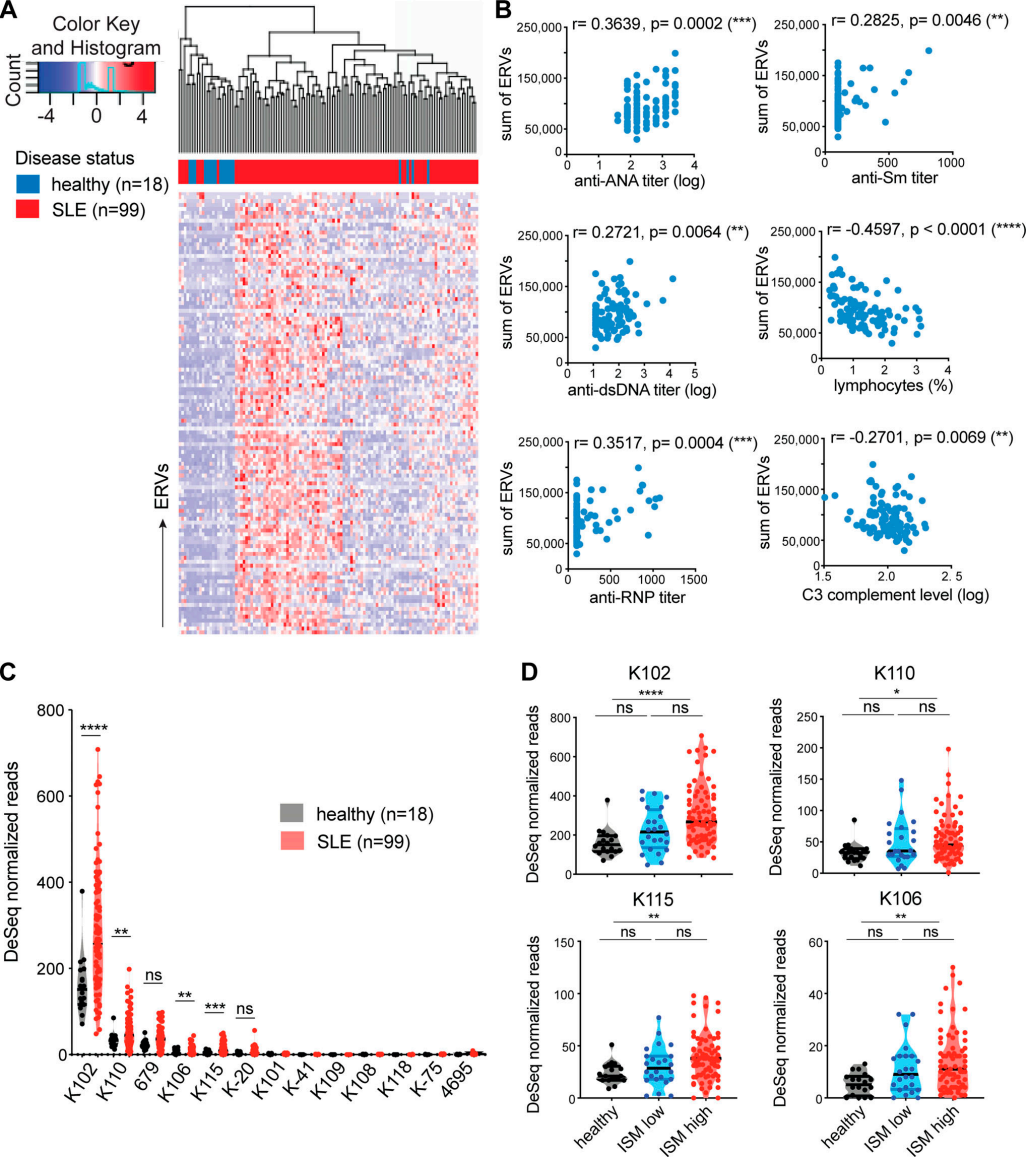
**Human-specific envelope-coding ERV-K loci are elevated in lupus blood.** ERVmap analysis of RNA-seq data from whole blood of healthy (*n* = 18) and SLE (*n* = 99) individuals was performed. **(A)** 113 significantly elevated ERV loci are depicted as a hierarchical cluster heatmap. **(B)** Spearman correlation between the sum of significantly elevated ERV read counts and levels of indicated clinical parameters. **(C)** Normalized read counts for ERV-K loci were compared between healthy and SLE samples. **(D)** Comparison of normalized ERV-K read counts between healthy (black), ISM low (blue), and ISM high (red) groups. Mann–Whitney *t* test was performed to calculate significance for C and D. *, P < 0.05; **, P < 0.01; ***, P < 0.001; ****, P < 0.0001. dsDNA, double-stranded DNA.

Using ERVmap, we observed a global elevation in proviral ERV expression in the whole blood of SLE patients compared with healthy controls in a published RNA-seq data from a cohort of SLE patients in the rontalizumab in SLE (ROSE) trial (Hung et al., 2015; Kalunian et al., 2016). Within this cohort, we again identified over 100 significantly elevated ERVs (Fig. 1 A). The total read counts from elevated ERVs significantly correlated with clinical parameters associated with disease, including titers of anti-nuclear antibody, anti-double stranded DNA, anti-ribonucleoprotein (anti-RNP), and anti-Sm antibodies as well as decreased lymphocyte counts and complement C3 levels (Fig. 1 B). These results showed a strong correlation between ERV signature and clinical indicators of SLE.

ERV-derived envelope (gp70) protein and immune complexes composed of gp70 protein are prevalent in lupus mouse models (Andrews et al., 1978; Izui et al., 1981). In addition, anti-gp70 immune complexes are known to mediate pathology in non-autoimmune mice (Andrews et al., 1978; Izui et al., 1981; Tabata et al., 2000). Based on these findings, we pursued the hypothesis that ERV-derived envelope proteins in humans have the potential to contribute to SLE and focused specifically on ERV-K (HML-2) members. In the ERVmap database, there are at least 87 ERV-K loci, and 12 of them encode an envelope protein without in-frame stop codons (Table S1). Based on ERVmap analysis, 4 out of the 12 ERV-K loci with envelope-coding sequences were significantly elevated in lupus blood compared with healthy controls: *K102*,* K106*,* K115*,**and *K110* (Fig. 1 C). The genomic locations and additional aliases associated with these loci are listed in Table S1.

**Figure S1.**
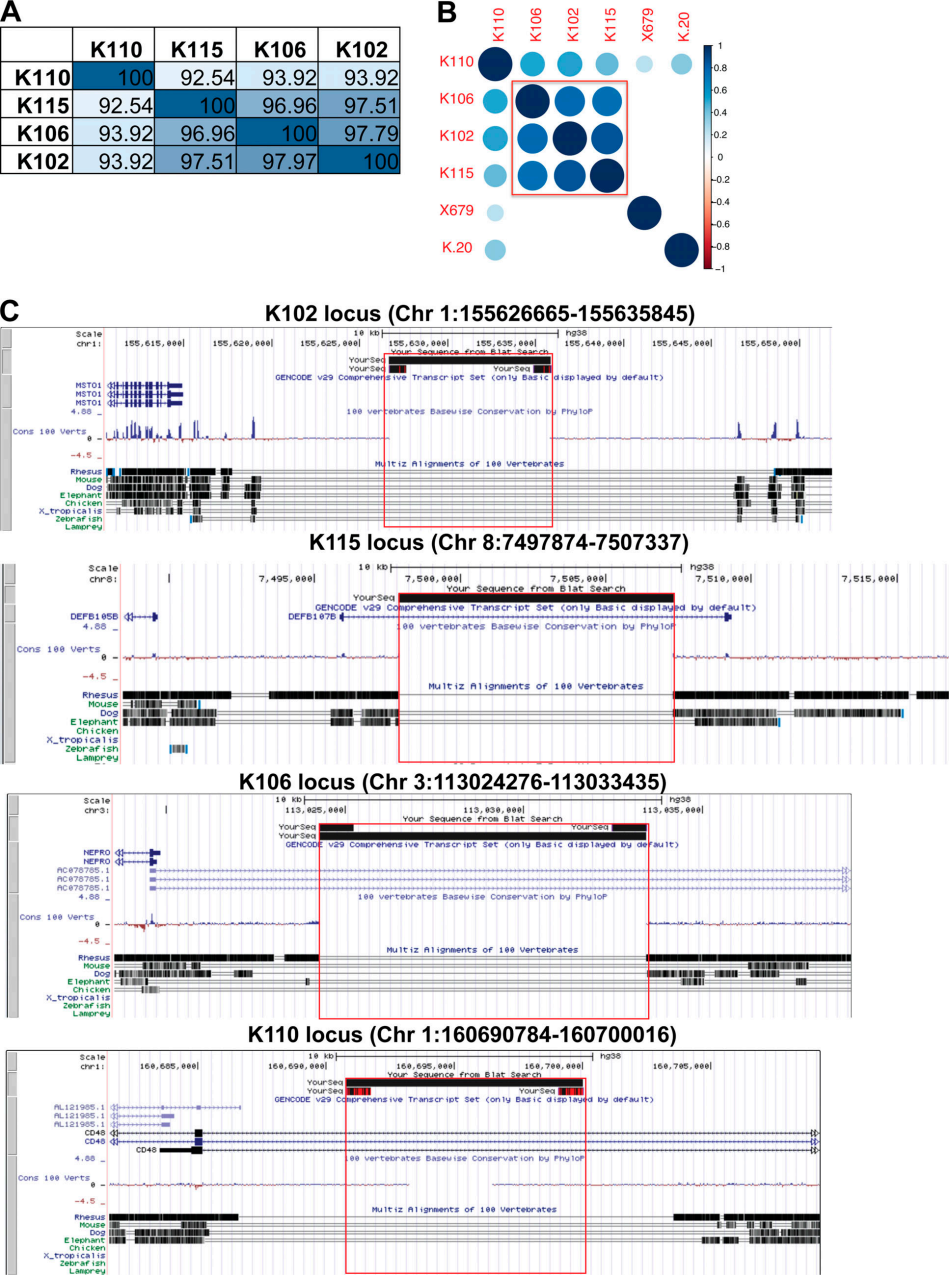
***ERV-K102***,***K115***,***K106*, and *K110* sequences are similar and human specific. (A)** Percent homology between the envelope SU sequences of the indicated ERV-K loci at the amino acid level. **(B)** Spearman correlation between normalized ERV-K read counts in SLE samples (*n* = 99). Scale bar represents Spearman r values and only showing correlations with P < 0.05. **(C)** UCSC genome browser outputs are displayed for each ERV-K locus to show absence of orthologous sequences in other vertebrate genomes. Red box indicates the full ERV-K locus. Chr, chromosome.

There were significant amino acid sequence homology between the envelope sequences of the four ERV-K loci, with up to 97% homology among *K102*,* K115*, and *K106* and 92% homology between *K110* and the other three ERV-K loci (Fig. S1 A). The expression levels of these loci correlated within individuals (Fig. S1 B), suggesting that these loci may be coregulated. Based on sequence annotation of these ERVs in the UCSC genome database, *K102*,* K115*,* K106*, and *K110* are human-specific ERVs, with no known homology to other primate genomes, and do not overlap with other gene loci (Fig. S1 C).

**Figure S2.**
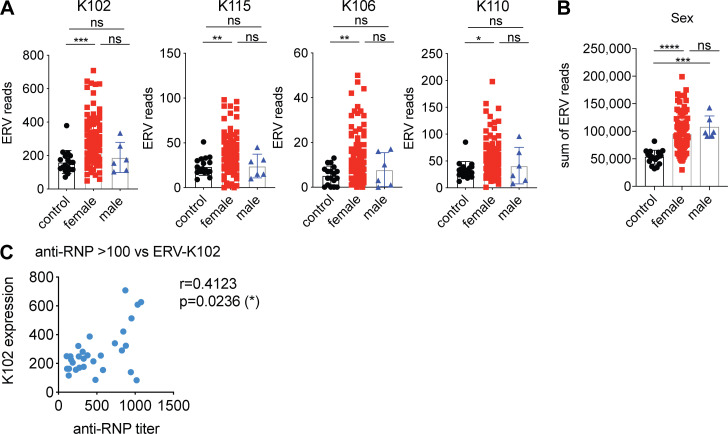
**ERV-K expression is higher in females than males and correlates with anti-RNP titer.** **(A and B)** Normalized ERV read counts for the indicated ERV-K loci (A) or sum of reads from the significantly elevated ERVs (B) were differentially plotted for females and males (control, *n* = 18; females, *n* = 93; males *n* = 6). Nonparametric one-way ANOVA analysis was performed to calculate statistical significance between groups. **(C)** Correlation plot of ERV-K102 read counts and anti-RNP titer for SLE patients with anti-RNP antibody titers over 100 (*n* = 30). *, P < 0.05; **, P < 0.01; ***, P < 0.001; ****, P < 0.0001.

The expression of *ERV-K102*,* K115*,* K106*, and *K110* were significantly elevated in female patients, but not in male patients (Fig. S2 A), even though total normalized ERV read counts did not significantly differ between female and male SLE patients (Fig. S2 B). In addition, *ERV-K102* expression in particular significantly correlated with anti-RNP titers, but not with other autoantibody levels (Fig. S2 C). Finally, we observed significantly higher expression of *ERV-K102*,* K115*,* K106*, and *K110* in patients with higher type I IFN signature metrics (ISMs; Fig. 1 D), supporting that ERV-K expression is associated with clinical indicators of SLE.

### Transcriptional regulators that correlate with expression of ERV-K102

ERV expression is regulated through epigenetic control, whereby DNA methylation and repressive histone methylation suppress expression of ERVs. These repressive marks on ERV loci are maintained by Kruppel-associated box domain zinc-finger proteins (KRAB-ZFPs)–TRIM28 complex and the human silencing hub (HUSH) complex (Ecco et al., 2016; Robbez-Masson et al., 2018). In SLE, epigenetic dysregulation is one of the hallmarks of disease (Ballestar and Li, 2017; Tsokos, 2011). Twin discordance in SLE is attributed to differences in DNA methylation, and enhanced expression of inflammatory genes in lupus CD4^+^ T cells is attributed to global hypomethylation (Javierre et al., 2010; Absher et al., 2013; Zhao et al., 2014). Hypomethylation at LTR2C in lupus CD4^+^ T cells is associated with enhanced *ERV-E* expression (Wu et al., 2015). Based on these previous studies, we sought to determine whether elevated ERV expression in the peripheral blood of lupus patients is a result of lower expression of known epigenetic silencers of ERVs.

**Figure 2.**
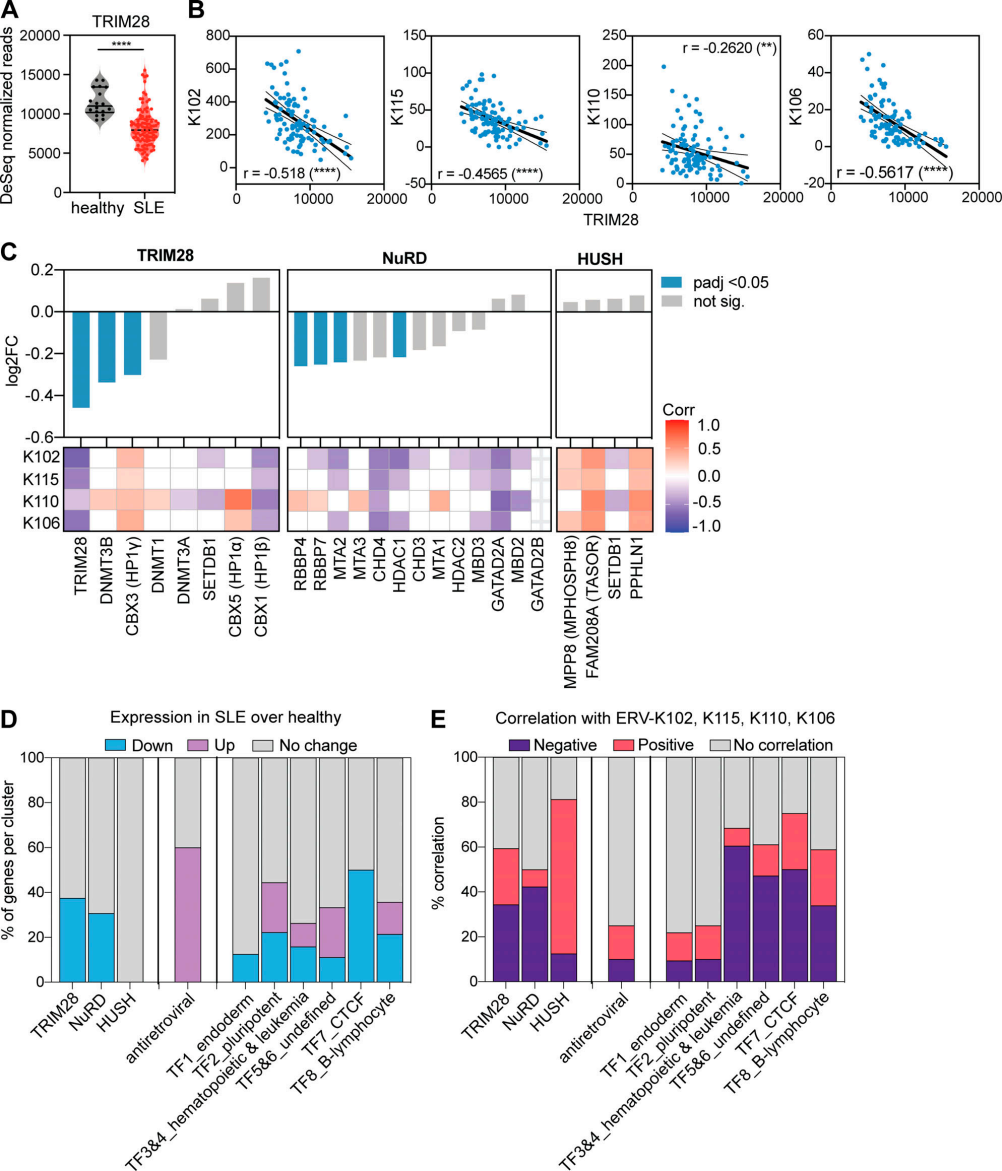
**Elevated *ERV-K* expression correlates with reduced epigenetic repressor expression.** Using the same RNA-seq dataset as the ERVmap analysis, the cellular transcriptome was analyzed. **(A)** Read counts for *TRIM28* in healthy donors (*n* = 18) and SLE patients (*n* = 99). Mann–Whitney *t* test was performed to calculate significance. ****, P < 0.0001. **(B)** Linear regression analysis on correlation between normalized read counts for *ERV-K102*,* K115*,* K106*, *K110*, and *TRIM28*. r, Pearson r. **(C)** Differential expression of the indicated epigenetic repressor genes between healthy donors and SLE patients grouped by repressor complexes. log2FC, log2 fold change of gene expression in SLE compared with healthy. Statistical significance (adjusted P value [padj]) calculated by DESeq2. Heatmap of correlation between *ERV-K* and indicated genes showing Spearman r values (1 to −1) for significant correlations (P < 0.05). White grids, no significant correlation. **(D)** Percent of genes within each cluster that are significantly different between healthy donors and SLE patients (DESeq2, padj < 0.05). **(E)** Percentage of genes that significantly correlate with *ERV-K* loci (Spearman correlation, P < 0.05).

Using cellular transcriptome data from the same RNA-seq dataset, we observed a significantly lower expression of *TRIM28* in SLE compared with healthy blood (Fig. 2 A) and a negative correlation between *ERV-K102*,* K115*,* K110*, and *K106* expression and *TRIM28* expression (Fig. 2 B); patients who expressed high levels of ERV-K expressed low levels of *TRIM28*. Similarly, we observed significantly lower expression of *DNMT3B* and *HP1γ*, which are part of the KRAB-ZFP–TRIM28 complex, and *RBBP4*,* RBBP7*,* MTA2*, and *HDAC1*, which are part of the nucleosome remodeling and deacetylase (NuRD) complex that associates with TRIM28 (Fig. 2 C). We did not observe a difference in the expression of the components of the HUSH complex. We also observed strong negative correlations between TRIM28 and NuRD complex and ERV-K expression, whereas HUSH complex positively correlated with ERV-K expression (Fig. 2 C).

Apolipoprotein B mRNA-editing enzyme catalytic polypeptide-like 3 (APOBEC3) family of proteins, tripartite motif–containing 5a (TRIM5a), and bone marrow stromal cell antigen 2 (BST2; tetherin) are well-established retroviral restriction factors that restrict HIV (Malim and Bieniasz, 2012) and ERVs in humans and mice (Goff, 2004; Anwar et al., 2013; Ganser-Pornillos and Pornillos, 2019; Treger et al., 2019a; Wolf and Goff, 2009). In addition, a number of transcription factors have been predicted to bind to the ERV LTR sequences (Manghera and Douville, 2013). Thus, we next expanded our analysis to additional factors that have been implicated in the regulation of ERVs.

**Figure S3.**
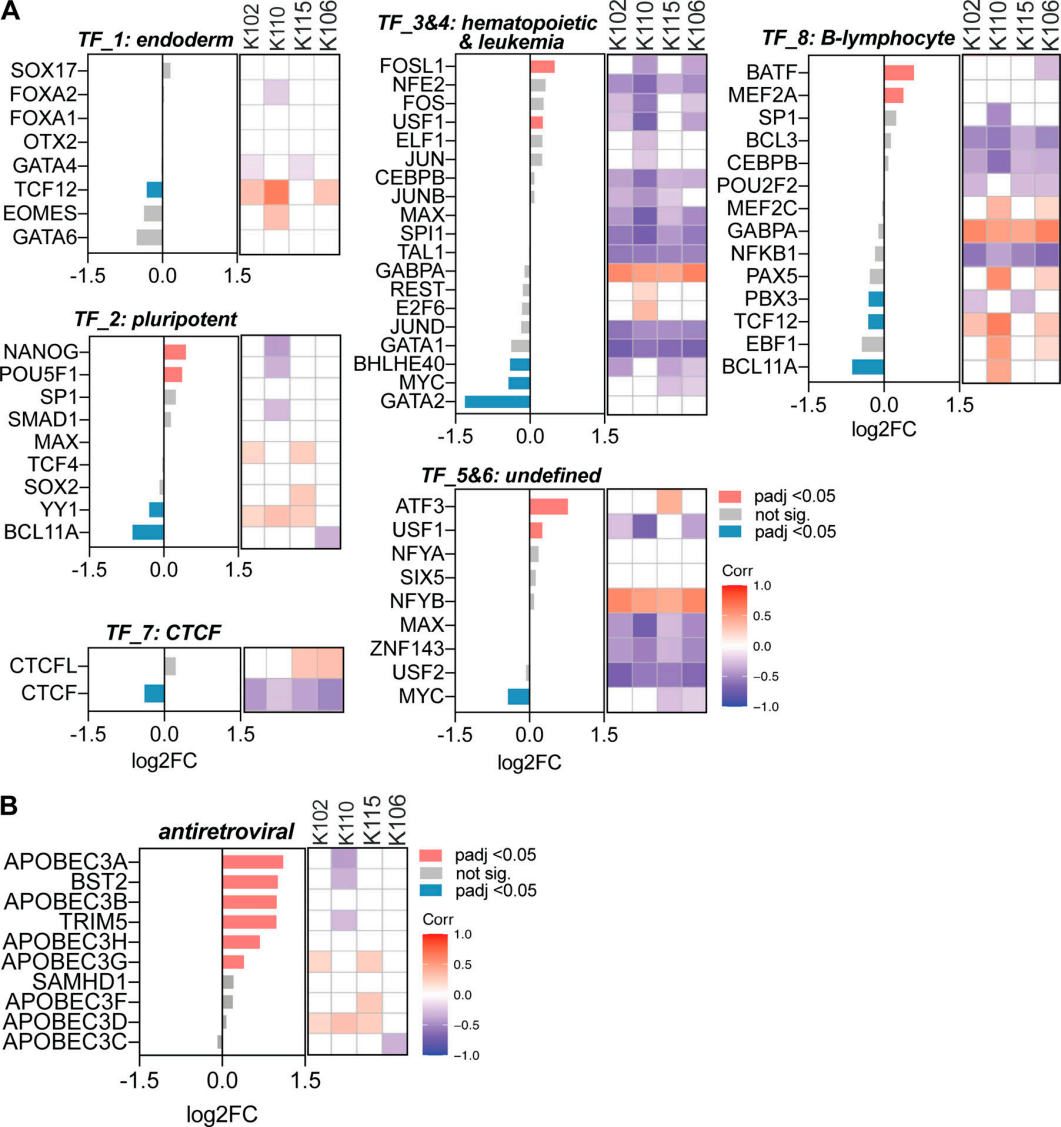
**Correlation between ERV-K expression and differentially expressed transcription factors and antiretroviral factors.** **(A and B)** Differential expression of genes in SLE over healthy (log2FC) for the indicated transcription factors (A) and antiretroviral factors (B). Differential expression and padj was determined using DESeq2 comparing SLE patients (*n* = 99) and healthy donors (*n* = 18). Spearman correlation between normalized ERV-K read counts versus indicated genes were plotted in R studio. Spearman *r* correlation values are colored according to the legend. Blank boxes, not significant. Corr, correlation; TF, transcription factor.

Overall, retroviral restriction factors were expressed higher in SLE patient blood compared with healthy controls, whereas expression of transcription factor clusters that were previously described for LTR elements varied (Fig. 2 D; and Fig. S3, A and B; Ito et al., 2017). There was very little correlation between retroviral restriction factors and ERV-K expression, but transcription factors within clusters 3–7 negatively correlated with ERV-K expression (Fig. 2 E; and Fig. S3, A and B). These data suggest that the loss of epigenetic silencing machinery expression has a stronger association with elevated ERV expression than expression of LTR-regulating transcription factors or retroviral restriction factors in SLE.

### Cloning and generation of recombinant ERV-K102 envelope SU protein

**Figure 3.**
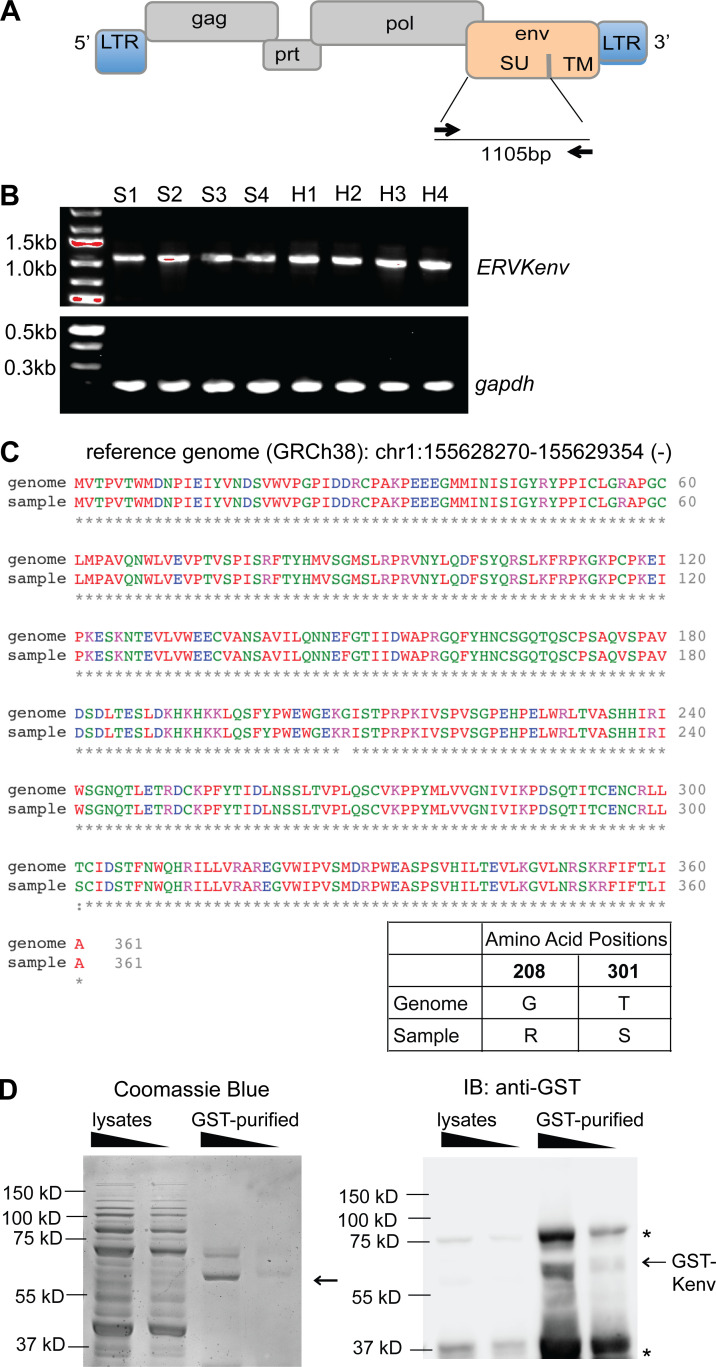
**Generation of recombinant envelope protein encoded by the *ERV-K102* locus. (A)** Schematic representation of the proviral structure of ERV-K sequence and the positions for the primers to amplify the SU of the envelope are indicated as arrows. TM, transmembrane. **(B)** DNA agarose gels of products from PCR amplification of ERV-K envelope SU and *gapdh* from healthy (H; *n* = 4) and SLE (S; *n* = 4) PBMC cDNA. **(C)** Amino acid sequence alignment between the reference ERV-K102 sequence (hg38) and the dominant product amplified from PBMC cDNA. Amino acid differences at positions 208 and 301 are depicted. **(D)** GST-purified recombinant ERV-K102 envelope SU analyzed by Coomassie blue staining and Western blot with anti-GST antibody. Asterisks indicate nonspecific bands. IB, immunoblot.

**Figure S4.**
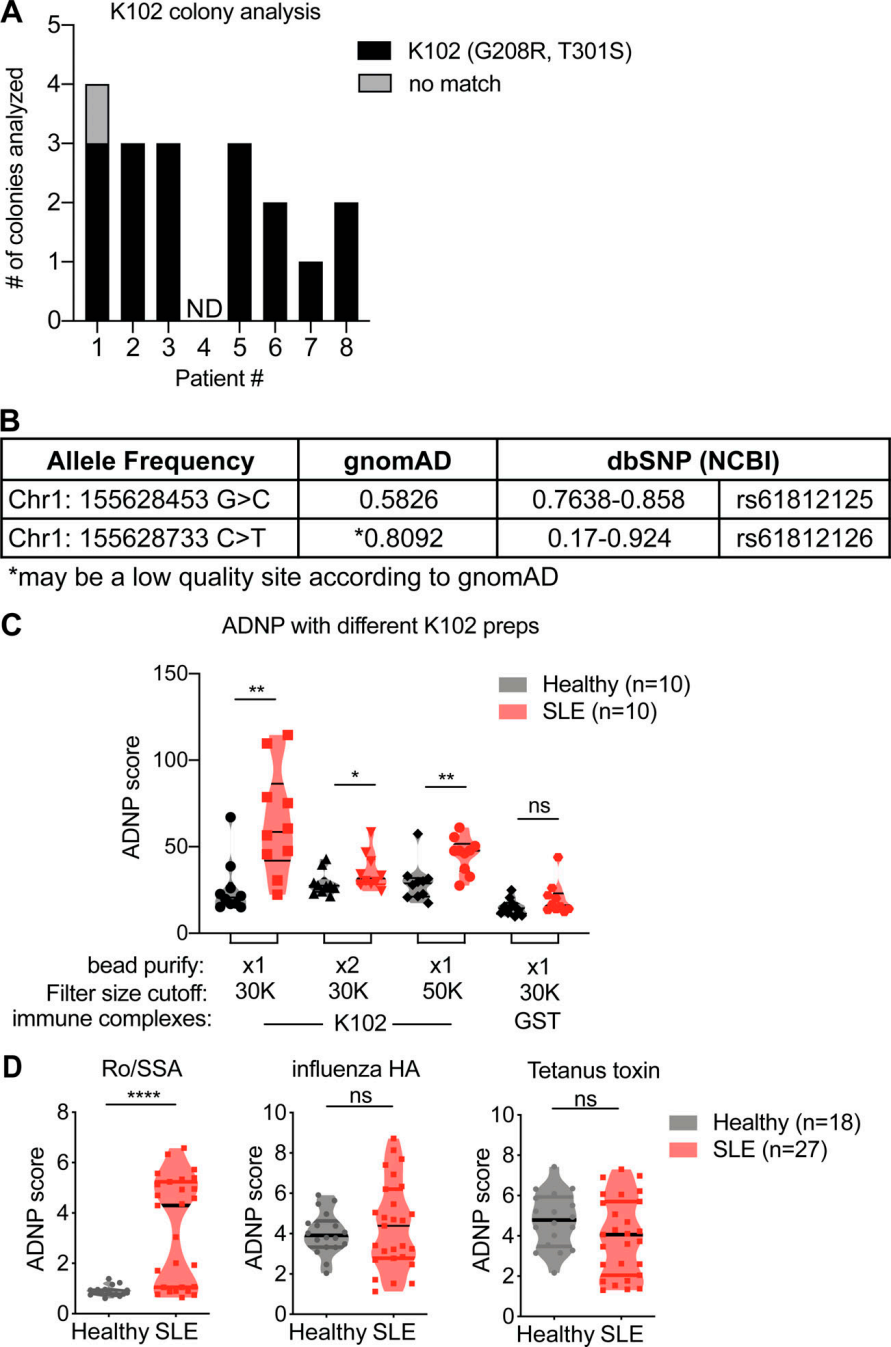
**ERV-K102 cDNA sequence analysis and ADNP with different K102 protein preps and other antigens. (A)** Sequencing analysis of ERV-K102 DNA amplified from SLE PBMC cDNA and cloned into a sequencing vector. BLAT results against for each of the inserts against hg38 per SLE patient (*n* = 8). No match, no alignment between insert and hg38. **(B)** Summary of the allele frequency for Chr1: 155628453 G>C (T301S) and Chr1: 155628733 C>T (G208R). **(C)** ADNP assay with immune complexes containing recombinant ERV-K SU envelope protein that were purified through an additional round of GST-bead purification or larger cutoff spin columns. Purified GST was used as a negative control. **(D)** ADNP assay with immune complexes containing the indicated antigens and healthy or SLE plasma. Mann–Whitney *t* test was performed to calculate statistical significance. *, P < 0.05; **, P < 0.01; ****, P < 0.0001.

To confirm our RNA-seq data, we used a previously described approach to amplify the surface unit (SU) of ERV-K envelope sequences (Wang-Johanning et al., 2001) from the cDNA of healthy and SLE PBMCs (Fig. 3 A). We observed a 1,105-bp band, as expected, in both healthy and SLE PBMC samples (Fig. 3 B). We next cloned the PCR products from eight SLE patient samples into a sequencing vector and sequenced the inserts by Sanger sequencing. We detected one dominant product from all samples, and basic local alignment search tool (BLAST)–like alignment tool (BLAT) analysis against the hg38 human genome revealed that it is derived from the anti-sense strand of chromosome 1 between 155628270 and 155629354 (1q22), which belongs to the *K102* locus, supporting our results from the ERVmap analysis (Fig. S4 A). The amino acid sequence of the cloned K102 product was nearly identical to the reference genome except for two mutations at G208R and T301S (Fig. 3 C). Based on gnomAD and dbSNP reports by the National Center for Biotechnology Information, variants at these sites (Chr1: 155628453 [T301S, G>C], Chr1:155628733 [G208R, C>T]) are common genomic variants (Fig. S4 B). To study the role of this envelope protein in lupus, we cloned an N-terminal glutathione-S-transferase (GST)–tagged 1,086-bp SU of the envelope protein beginning at the internal methionine, which generates a coding sequence without stop codons and is identical to the sequence in the human proteome database (NX_P61567). The production of a 65-kD protein after GST-bead purification was observed by both Coomassie blue staining and anti-GST immunoblot (Fig. 3 D).

### Levels of anti-ERV-K102 IgG in SLE patients correlate with ISG expression

**Figure 4.**
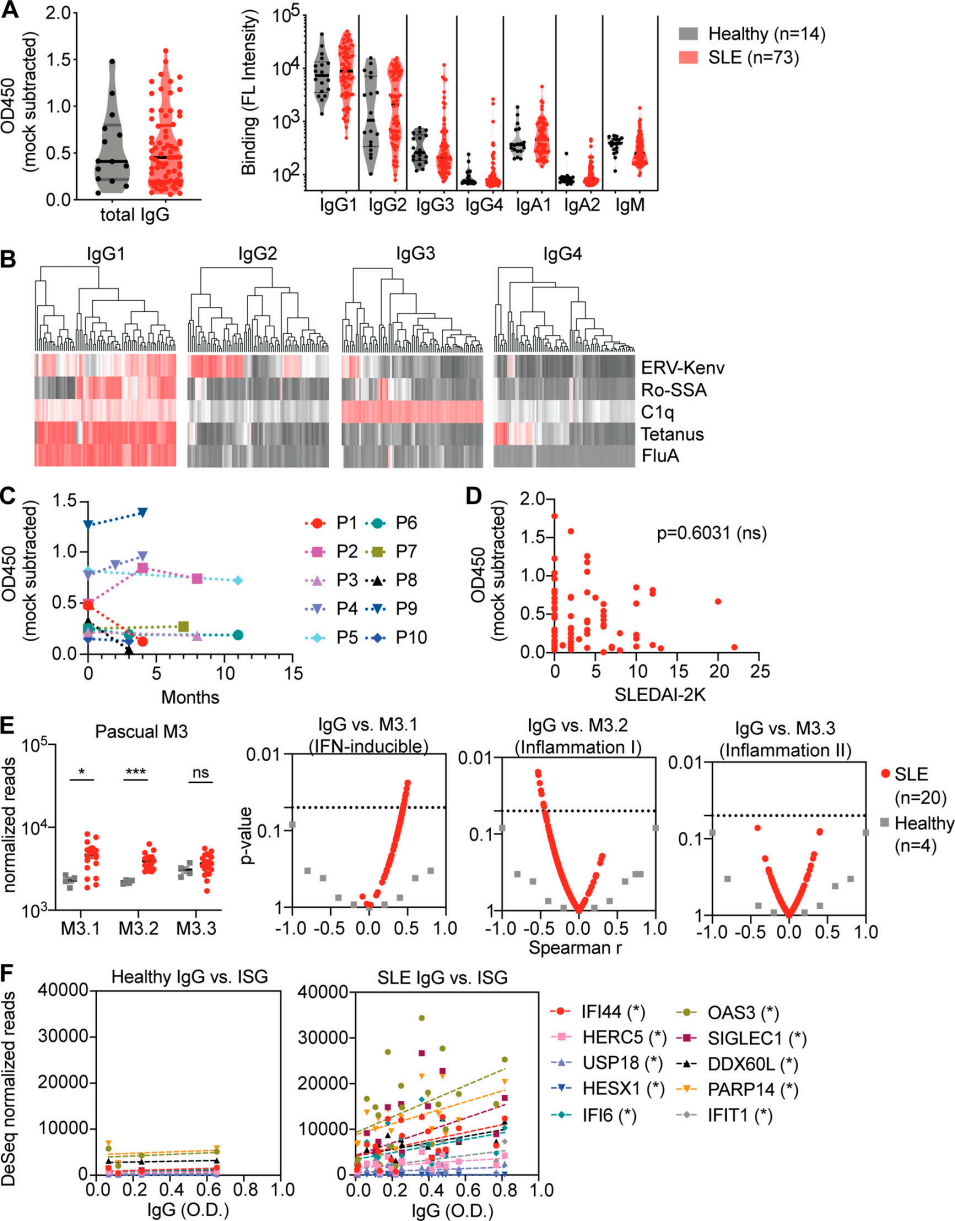
**Neutrophil activation by SLE IgG in an immune complex with ERV-K102 envelope protein. (A)** Total IgG against recombinant ERV-K102 envelope SU measured by ELISA and IgG subclasses measured by Luminex assay in healthy (*n* = 14) and SLE (*n* = 73) plasma. **(B)** Hierarchical clustering of IgG levels in SLE patients for the indicated antigens and IgG subclasses measured by Luminex assay. FL, fluorescence. **(C)** Total anti-ERV-K102 IgG levels in SLE patients over the indicated months. Each line represents an individual patient (P; *n* = 10). **(D)** Correlation between anti-ERV-K102 IgG levels as measured by ELISA and SLEDAI-2K score obtained at the time of blood collection for SLE patients (*n* = 79). Spearman correlation analysis was performed. **(E)** Comparison of read counts for genes within M3.1, 3.2, and 3.3 modules between healthy (*n* = 4) and SLE (*n* = 20). Mann–Whitney *t* test was performed to calculate significance (*, P < 0.05; ***, P < 0.001). Volcano plot of Spearman r values obtained from correlation analysis between anti-ERV-K102 IgG levels and total read counts for genes in the indicated modules. Each dot represents an individual and dotted line is at P = 0.05. **(F)** Spearman correlation between read counts for the indicated genes versus total anti-ERV-K102 IgG levels measured by ELISA. *, P < 0.05.

We next investigated whether SLE patients possess antibodies against ERV-K102 envelope protein. We measured both total IgG and IgG subclasses against the recombinant ERV-K102 envelope SU in healthy and SLE plasma using ELISA and Luminex assay, respectively. We observed comparable levels of total anti-ERV-K102 IgG and subclass-specific IgG against ERV-K102 in both healthy and SLE plasma (Fig. 4 A). IgG1 and IgG2 reactivity was observed against ERV-K102 envelope compared with known autoantigens and vaccine antigens like tetanus toxin and influenza hemagglutinin (HA) protein, for which IgG1 reactivity was dominant (Fig. 4 B). We found that anti-ERV-K102 IgG levels were relatively stable in SLE patients over a 6- to 10-mo period (Fig. 4 C). To probe whether varying levels of anti-ERV-K102 IgG among SLE patients correlate with disease severity, we first compared anti-ERV-K102 IgG with SLE disease activity index (SLEDAI)–2K score (Gladman et al., 2002) and found no significant correlation (Fig. 4 D). We next compared anti-ERV-K102 IgG levels to inflammatory gene expression in our smaller Yale cohort of SLE patients using previously established gene modules (Chaussabel et al., 2008). M3.1 (IFN-inducible genes) and M3.2 (inflammation I) modules were significantly elevated in SLE compared with healthy controls (Fig. 4 E). Significant correlation was observed between anti-ERV-K102 IgG levels and the IFN-inducible gene module (M3.1), but not with M3.2 or M3.3 (Fig. 4 E). In support of this, anti-ERV-K102 IgG levels significantly correlated with the expression of IFN-stimulated genes (ISGs) in SLE patients, but not healthy donors (Fig. 4 F). Together, our data showed that, despite comparable levels of anti-ERV-K102 IgG between healthy donors and SLE patients, the latter significantly correlated with IFN-inducible gene expression and suggested that anti-ERV-K102 IgG from SLE patients may functionally differ from healthy IgG.

### Anti-ERV-K102 IgG from SLE patients activate neutrophils in the form of immune complexes with ERV-K102 envelope

Immune complexes containing self-antigens are major sources of inflammation in autoimmune diseases and act through binding to Fc receptors on innate immune cells. Neutrophils are the most abundant immune cell type in the blood and play a central role in the pathogenesis of lupus disease. Neutrophils are activated by autoantibody immune complexes, and upon activation, secrete intracellular nucleic acids bound by anti-microbial peptides through neutrophil extracellular traps (NETs; Lande et al., 2011; Garcia-Romo et al., 2011; Kaplan, 2011; Yu and Su, 2013; Thieblemont et al., 2016).

**Figure 5.**
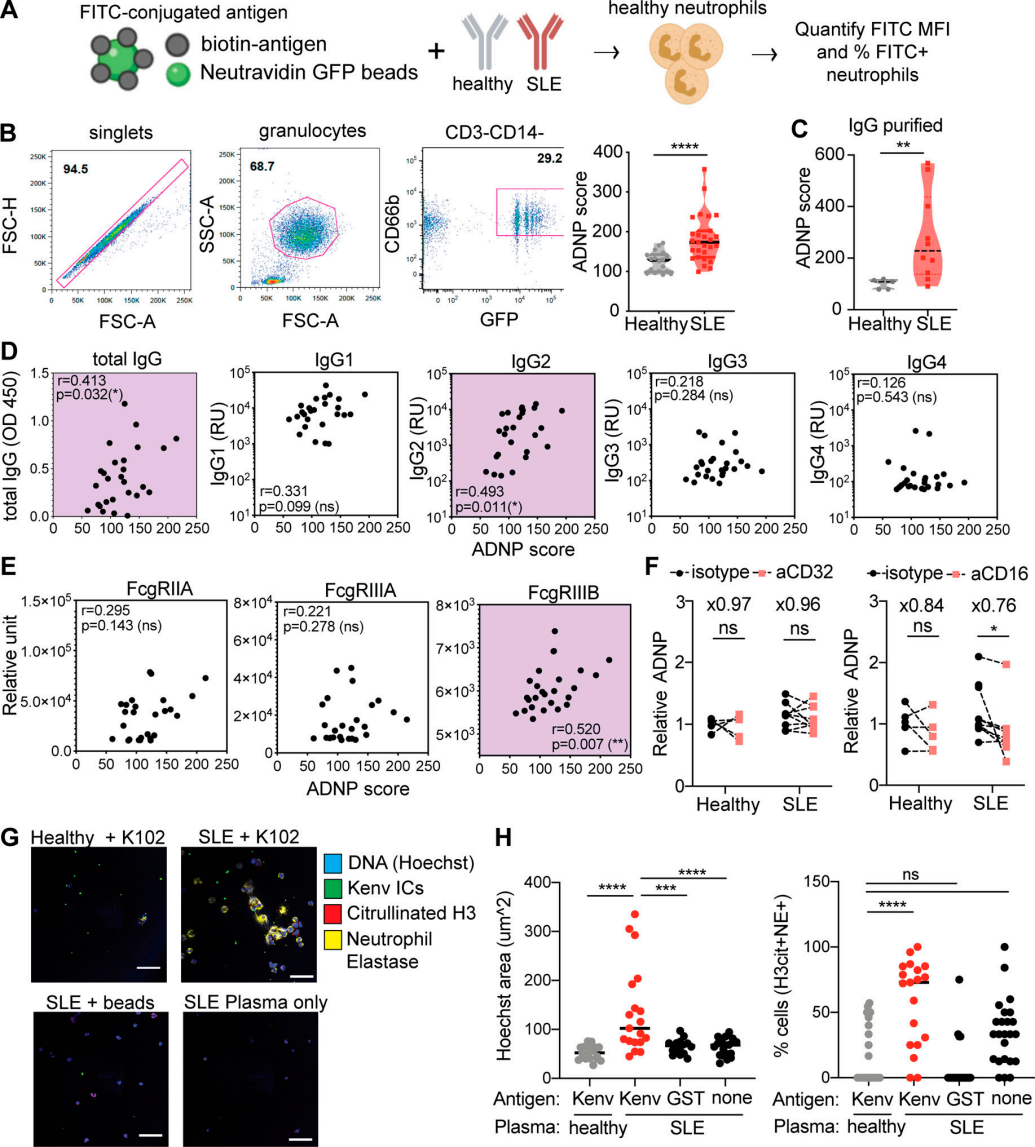
**ERV-K102 envelope immune complexes with SLE IgG induce higher neutrophil phagocytosis and neutrophil activation.** **(A)** ADNP assay scheme. **(B)** Gating strategy for quantification of ADNP by flow cytometry and ADNP of healthy (*n* = 18) and SLE (*n* = 27) immune complexes. **(C)** ADNP of ERV-K102 immune complexes made with purified IgG from healthy and SLE plasma. Significance was calculated using the Mann–Whitney *t* test. **, P < 0.01; ****, P < 0.0001. **(D and E)** Spearman correlation between anti-ERV-K102 IgG levels (D) or ERV-K102 immune complex binding to FcγRs (E) and ADNP for SLE samples (*n* = 26). *, P < 0.05; **, P < 0.01. **(F)** ADNP of ERV-K102 immune complexes (healthy, *n* = 5; and SLE, *n* = 10) in neutrophils pretreated with isotype IgG, anti-CD32, or anti-CD16 IgG. Relative ADNP was calculated based on the average ADNP score for healthy samples. Average fold difference between isotype- and specific antibody–treated samples is indicated above each condition. Mann–Whitney *t* test was used to calculate statistical significance. *, P < 0.05. **(G)** Representative confocal images of neutrophils treated with ERV-K102 immune complexes or indicated controls for healthy (*n* = 5) and SLE (*n* = 5) plasma. Hoechst (blue), citrullinated histone H3 (red), neutrophil elastase (yellow), and FITC-conjugated immune complexes (green) are shown. Data are representative of two or more repeated experiments. **(H)** Area of Hoechst nuclear DNA staining (square millimeters) was measured per cell in ImageJ. The average area of four cells per image is plotted. For each condition for each donor plasma, four images were recorded, and four cells were measured per image. Scale bars, 75 µm. The percentage of neutrophils that costained with of citrullinated histone H3 and neutrophil elastase per image was calculated. One-way ANOVA with multiple comparisons was performed to calculate statistical significance. ***, P < 0.001; ****, P < 0.0001. FSC-A, forward scatter A; FSC-H, forward scatter H; IC, immune complex; SSC-A, side scatter A.

Given that anti-ERV-K102 IgG levels correlated with ISG expression, we tested whether immune complexes containing ERV-K102 envelope protein and anti-ERV-K102 IgG have the potential to activate neutrophils. To test this, we generated immune complexes using recombinant ERV-K102 envelope SU protein conjugated to FITC beads and incubated with either healthy or SLE plasma. We then cultured the immune complexes with primary neutrophils isolated from healthy donors and measured immune complex phagocytosis by flow cytometry. We calculated antibody-dependent neutrophil phagocytosis (ADNP) score based on the percentage of FITC^+^CD14^−^CD66b^+^ neutrophils and mean fluorescence intensity (MFI) of FITC (Fig. 5 A; Gunn et al., 2018).

We observed enhanced neutrophil phagocytosis of ERV-K102 immune complexes generated with SLE plasma compared with healthy plasma (Fig. 5 B). This enhancement was observed even with purified IgG (Fig. 5 C), excluding the involvement of other plasma proteins. Enhanced ADNP was also observed using immune complexes generated with K102 envelope SU protein that was further purified (Fig. S4 C). We independently generated immune complexes with known autoantigen (Ro/SSA) and common vaccine antigens (tetanus toxin and influenza HA). Although the extent of ADNP for each immune complex varied depending on the donor neutrophils, we observed enhanced neutrophil phagocytosis of anti-Ro/SSA immune complexes with SLE plasma, as expected due to higher levels of anti-Ro/SSA IgG in SLE patients. We did not observe differences in neutrophil phagocytosis of tetanus toxin immune complexes or influenza HA immune complexes between healthy and SLE plasma (Fig. S4 D), suggesting that enhanced neutrophil phagocytosis is not a general feature of SLE IgG immune complexes but rather specific to immune complexes containing autoantigens.

**Figure S5.**
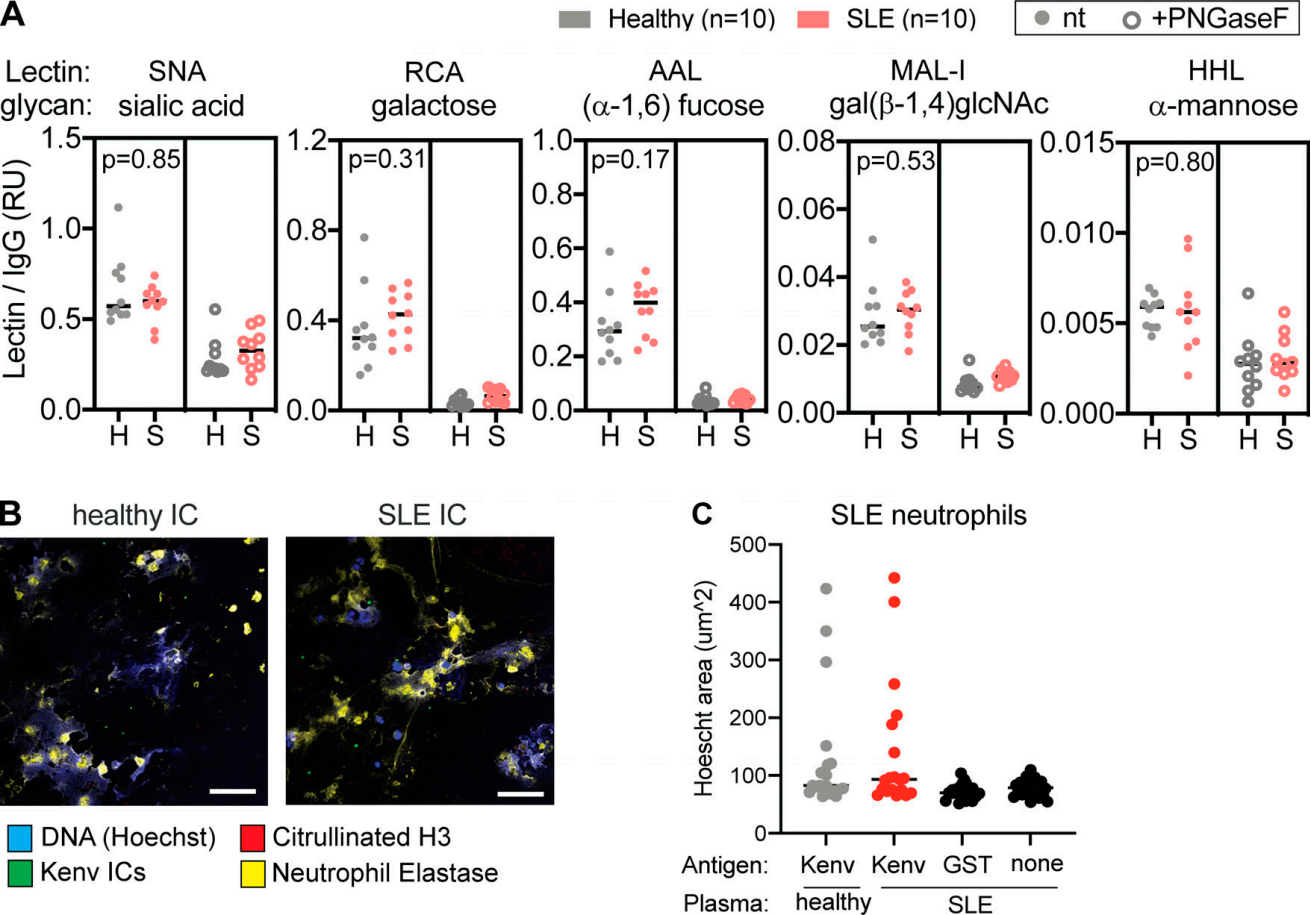
**Glycan modifications of anti-ERV-K102 IgG and NETosis in SLE neutrophils. (A)** Glycan modifications on anti-ERV-K102 IgG (healthy and SLE) detected using biotinylated lectins and quantified by a Luminex assay. Lectin binding was quantified for untreated IgG (left panel) and PNGase F–treated IgG (right panel). Relative units were calculated by normalizing MFI of each lectin signal by MFI of total human IgG for each sample. Mann–Whitney *t* test was performed to calculate statistical significance. *, P < 0.05; **, P < 0.01. **(B)** Representative microscopy images of neutrophils from SLE patients stimulated with ERV-K102 immune complexes (ICs) generated with healthy plasma (*n* = 5) or SLE plasma (*n* = 5). **(C) **NETs were quantified by measuring the area of Hoechst staining per cell in ImageJ. For each condition per donor plasma, four images were recorded, four cells were measured per image, and the average area per image was plotted. Scale bars, 75 µm. RU, relative unit.

We next considered whether enhanced neutrophil phagocytosis was reflective of anti-ERV-K102 IgG levels within SLE samples. We found that indeed ADNP significantly correlated with the levels of total anti-ERV-K102 IgG and more specifically with the levels of anti-ERV-K102 IgG2 in SLE (Fig. 5 D). This implies that although ERV-K102 IgG levels were not significantly higher in SLE compared with healthy controls, anti-ERV-K102 IgG in SLE mediates enhanced ADNP when complexed with the K102 antigen. We next found that ADNP correlated with binding of ERV-K102 immune complexes to FcγRIIIB, a low-affinity receptor for IgG2 and the most abundantly expressed FcγR on neutrophils (Fig. 5 E; Bruhns, 2014). As antibody glycosylation is a key determinant of FcγR binding (Gunn and Alter, 2016), we further assessed glycosylation of anti-ERV-K102 IgG specifically. We found that fucosylation, which dictates FcγRIIIB binding, was slightly elevated in SLE patients, albeit not significantly, and other modifications were comparable between SLE and healthy IgG (Fig. S5 A). Blocking FcγRIII using anti-CD16 antibody resulted in a partial reduction of ADNP of ERV-K102 immune complexes (Fig. 5 F), indicating that enhanced neutrophil phagocytosis of ERV-K102 immune complexes with SLE plasma likely involves binding to FcγRIIIB on neutrophils.

Finally, we tested whether neutrophil phagocytosis of ERV-K102 immune complexes results in neutrophil activation and secretion of intracellular DNA in the form of NETs, a prominent inflammatory feature associated with SLE. We incubated healthy neutrophils with ERV-K102 immune complexes generated with either healthy or SLE plasma and stained cells for DNA, citrullinated histone H3, and neutrophil elastase. We observed large areas of extracellular DNA and coexpression of citrullinated histone H3 and neutrophil elastase when neutrophils were cultured with SLE immune complexes containing ERV-K102 envelope protein, but not when cultured with SLE plasma alone, healthy immune complexes, or immune complexes generated with GST (Fig. 5, G and H). These features are consistent with NETs previously characterized in SLE (Garcia-Romo et al., 2011; Lande et al., 2011; Villanueva et al., 2011). We further showed that neutrophils from SLE patient blood also form NETs in response to ERV-K102 immune complexes made with SLE IgG, although they are similarly responsive to ERV-K102 immune complexes made with healthy plasma (Fig. S5, B and C). Together, our data show that ERV-K102 immune complexes formed with SLE IgG are readily phagocytosed by neutrophils and induce NET formation.

## Discussion

ERVs are increasingly associated with diseases ranging from cancer, neurodegenerative diseases, and HIV (Rooney et al., 2015; Schmitt et al., 2013a, 2013b; Michaud et al., 2014; Li et al., 2015). In SLE, studies have reported association between ERVs and disease, but they have been limited to a small number of ERV loci (Ogasawara et al., 2000; Fali et al., 2014; Perl et al., 1995). While ERVs have been implicated in lupus pathogenesis (Perl et al., 2008; Nelson et al., 2014; Yu, 2016; Mellors and Mellors, 1976), the mechanisms by which ERVs potentially contribute to systemic inflammation in SLE remain ill-defined. In this study, we used ERVmap to expand our understanding of ERVs in SLE and characterized the inflammatory potential of anti-ERV-K envelope IgG.

We previously showed that ERV expression is elevated in PBMCs of SLE patients from a New Haven cohort (Tokuyama et al., 2018). In the present study, we examined RNA-seq data from an independent cohort of SLE patients from the ROSE trial (Kalunian et al., 2016). Consistent with our previous study, we found over 100 ERV loci that are elevated in the blood of SLE patients in this cohort. ERV expression levels positively correlated with disease markers such as elevated autoantibodies, decrease in complement proteins, and heightened IFN signature. The current data support our previous work showing that indeed ERV expression is broadly elevated in the peripheral blood of SLE patients and further revealed relevance to disease. In addition, our transcriptome-based ERV analysis expands the scope of ERV dysregulation in SLE beyond the well-studied HRES-1 p28 (Banki et al., 1992; Perl et al., 1995).

We found that elevated ERV expression in SLE is associated with reduced expression of epigenetic repressors of ERVs. This is consistent with a previous study showing that DNA hypomethylation of ERV-E and ERV-K LTRs in T cells corresponds to increased ERV expression (Nakkuntod et al., 2013) and our work showing a negative correlation between KRAB-ZFP expression and ERV expression in SLE blood (Treger et al., 2019b). Given that an altered epigenome is a key initiating event in SLE disease (Tsokos et al., 2016; Ballestar and Li, 2017; Hedrich and Tsokos, 2011), elevated ERV expression may, in part, be a consequence of altered epigenomes in these patients. Future experiments will seek to identify the exact factors regulating ERV expression in SLE pathogenesis.

We showed that an envelope protein encoded by one of the ERV-K (HML-2) loci, K102, is targeted by antibodies from SLE patients, and immune complexes formed with SLE IgG containing K102 envelope are readily phagocytosed by neutrophils. Despite comparable levels of anti-ERV-K102 envelope IgG in healthy donors and SLE patients, we observed enhanced neutrophil phagocytosis of immune complexes formed with SLE IgG. We found that this is partially explained by enhanced binding of immune complexes to FcγRIIIB. In SLE, mature neutrophils in the peripheral blood are distinguished from immature neutrophils by the expression of FcγRIIIB and show gene signatures of higher activation and type I IFN response (Mistry et al., 2019). Although we did not directly measure the downstream consequences of FcγRIIIB engagement by ERV-K immune complexes, enhanced uptake of immune complexes by FcγRIIIB may potentiate inflammation in SLE. To the best of our knowledge, our study is the first to show neutrophil activation by ERV-K envelope immune complexes in the context of disease.

Beyond the role of ERV-K102 SU in immune complex–mediated inflammation, there are other potential consequences of elevated expression of this locus. ERV-K102 is one of the hominoid-specific ERVs composed of LTR5_Hs sequence that is predominantly associated with younger ERVs. Although infectious ERVs have not been detected in humans, the human tetracarcinoma cell line Tera-1 produces virions from ERV-K loci, including ERV-K102 (Löwer et al., 1993; Bhardwaj et al., 2015). Viral-like particles have also been detected in ERV-K–expressing human blastocytes (Grow et al., 2015). These studies point to the possibility that viral-like particles of ERV-K102 may arise under disease conditions like lupus.

Our study demonstrated that ERV-K envelope is a target of autoantibodies in SLE, and anti-ERV-K envelope immune complexes are capable of mediating neutrophil activation and NET formation. Given the key role of neutrophils in SLE disease (Kaplan, 2011) and promoting the IFN cascade (Crow, 2014), ERV immune complexes may contribute to inflammation in SLE. Future studies should further reveal the contribution of ERV antigens and ERV immune complexes in systemic inflammation in SLE. While ERVs have been previously implicated in SLE, our study demonstrates the power and potential of ERVmap to systematically uncover novel mechanistic insights into the role of ERVs in autoimmunity.

## Materials and methods

### Patient information

Blood from SLE patients was obtained from two different cohorts. One cohort was recruited from the rheumatology clinic of Yale School of Medicine and Yale New Haven hospital in accordance with a protocol approved by the institutional review committee of Yale University (#0303025105). The diagnosis of SLE was established according to the 1997 update of the 1982 revised American College of Rheumatology criteria (Hochberg, 1997; Tan et al., 1982). After obtaining informed consent, peripheral blood was collected in EDTA tubes from human subjects, and plasma was extracted upon centrifugation. Plasma were stored at −80°C. Samples from a cohort in the SLE Biorepository at Brigham Women’s Hospital was also obtained. Institutional review board–approved consented whole blood samples were obtained from patients followed in the Brigham and Women’s Hospital Lupus Center (Brigham and Women’s Lupus Center Biobank institutional review board no. 2008P000130). All patients had SLE according to the American College of Rheumatology criteria for classification of SLE. Data were collected on age at diagnosis, current age, current SLE disease activity by the SLEDAI (Lam and Petri, 2005), disease manifestations, past medical history, and past and current medications.

Healthy donor samples were obtained at Yale University School of Medicine in accordance with a protocol approved by the institutional review committee of Yale University (no. 0409027018). Inclusion criteria for healthy volunteers included age 21–40 yr or ≥65 yr and ability to understand and give informed consent in English. Exclusionary criteria included current use of medication (such as antibiotics) in past 2 wk, evidence of acute infection (identified by self-report of fever or symptoms 2 wk before blood draw), and treatment for cancer in the past 3 mo. At screening (by self-report) women who were pregnant or possibly pregnant were excluded. Patients with the following medical history were also excluded: history of organ, bone marrow, or stem cell transplant, liver cirrhosis, kidney disease requiring dialysis, positive for HIV/AIDs, hepatitis C, or active hepatitis B, blood donation of 1 pint or more in the past 2 mo, or treatment with clinical trial medication.

Clinical data for SLE patients, including baseline levels of anti-nuclear antibody, anti-double stranded DNA, anti-Sm, anti-RNP, anti-La antibodies, lymphocyte counts, and complement levels, were obtained as part of the ROSE trial (Kalunian et al., 2016) and shared through an agreement with Genentech.

### RNA-seq analysis

RNA-seq data from healthy donor and SLE patient whole blood were obtained from a published source (Gene Expression Omnibus: GSE72509; PRJNA294187; Hung et al., 2015). Reads were aligned to the human genome (GRCh38), and ERVmap analysis and cellular gene analysis were performed according to previously described methods (Tokuyama et al., 2018). As described previously, ERV read counts were normalized to size factors obtained through cellular transcriptome analysis, and the normalized counts were used for all subsequent data analysis. Bioconductor R software was used to generate heatmaps, Spearman correlation plots, and star plots (RStudio Team, 2020).

### Cloning of ERV-K envelope

PBMCs from Yale healthy donors and SLE patients were obtained through Ficoll-Paque density centrifugation separation. PBMCs were stored in Buffer RLT, and RNA was isolated according to manufacturer’s protocol (RNeasy kit; Qiagen). RT-PCR was performed to amplify ERV-K envelope using previously published primers (Wang-Johanning et al., 2001) and *GAPDH* using the following primers: ERV-K forward, 5′-AGA​AAA​GGG​CCT​CCA​CGG​AGA​TG-3′; ERV-K reverse, 5′-ACT​GCA​ATT​AAA​GTA​AAA​ATG​AA-3′; GAPDH forward, 5′-CAA​TGA​CCC​CTT​CAT​TGA​CC-3′; GAPDH reverse, 5′-GAC​AAG​CTT​CCC​GTT​CTC​AG-3′.

Amplified products of the expected size were extracted from agarose gels (Zymo Research) and ligated into pCR-Blunt II-TOPO vector for sequencing (Thermo Fisher). Sequencing analysis was performed using ApE software (http://jorgensen.biology.utah.edu/wayned/ape/), and alignment was performed using Clustal Omega (EMBL-EBI). ERV-K envelope SU was cloned out of the sequencing vector and ligated into pGEX-6p-1, a N-terminal GST-tag expression vector (GE Healthcare) between EcoRI and NotI sites, using the following primers: ERV-K EcoRI forward, 5′-ATC​GGA​ATT​CGT​AAC​ACC​AGT​CAC​ATG​GAT​GG-3′; ERV-K NotI reverse, 5′-ATC​GGC​GGC​CGC​TGC​AAT​TAA​AGT​AAA​AAT​GAA​TCT​TTT​GGA​TCT​A-3′.

### Recombinant protein generation and purification

A BL21 strain of *Escherichia coli* was transformed with ERV-K pGEX-6p-1 vector, and protein production and GST-bead purification were performed as previously described (Treger et al., 2019b). Transformed cells were grown overnight in YT medium (Sigma-Aldrich) containing ampicillin. Overnight culture was used to inoculate 1 liter of YT medium and grown until OD_600_ reached 0.6. Cells were cooled in ice-cold water for 10 min and grown for 16–18 h in 0.5 mM isopropyl-β-D-thiogalactoside at 16°C. Cells were pelleted, resuspended in lysis buffer (50 mM Tris, pH 7.4, 100 mM NaCl, 0.1% Triton X, 5 mM DTT, and protease inhibitor complete tablets) at a 1:20 ratio of lysis buffer to starting culture volume, freeze/thawed once, and sonicated in Bioruptor Plus TPX microtubes (Diagenode) for nine cycles of 30 s on and 30 s off. Clarified lysates were incubated with Glutathione Sepharose 4B resin (GE Healthcare Life Sciences) at a ratio of 1:40 (resin bed volume to lysate volume) for 2 h at 4°C on a rotator and washed three times in PBS containing protease inhibitor, and GST-tagged proteins were eluted three times with elution buffer (50 mM Tris-HCl, pH 8.0, 10 mM reduced glutathione, and protease inhibitor tablets) at a 1:1 ratio of bed volume to elution buffer volume. Eluted proteins were concentrated using Amicon Ultra 0.5-ml centrifugal filter tubes (nominal mol wt limit, 30 kD; Millipore) and quantified by NanoDrop Spectrophotometer (Thermo Fisher). Lysates and purified products were analyzed by acrylamide gel electrophoresis followed by standard Coomassie blue staining and Western blot analysis using a rabbit anti-GST-tag polyclonal antibody (CAB4169; Thermo Fisher).

### Immune complex generation

Immune complexes were generated using human plasma and recombinant protein as previously described (Gunn et al., 2018). Ro-SSA antigen was purchased (Arotec Diagnostics), and HA and tetanus proteins were obtained from ImmuneTechnology and MasBiologics, respectively. Recombinant proteins were biotinylated with EZ-Link sulfo-NHS-LC-LC biotin (Thermo Fisher) at a 50 M excess for 30 min at room temperature. Excess biotin was removed using 7K MWCO Zeba Spin Desalting Columns (Thermo Fisher) according to the manufacturer’s protocol. Biotinylated proteins were coupled to FITC-labeled 1-µm FluoSpheres NeutrAvidin-labeled Microspheres (Thermo Fisher) at a 1 μg to 1 μl ratio of protein to beads for 2 h at 37°C, washed twice in 0.1% BSA in PBS, and resuspended in 1 ml of 0.1% BSA in PBS for 10 μg protein. 0.1 μg bead-coupled proteins was incubated with 100 μl of 1:100 dilution of plasma IgG in a 96-well plate for 2 h at 37°C to generate immune complexes, and beads were pelleted by centrifugation at 2,000 rpm for 10 min. For IgG purification, plasma was diluted 1:10 in Melon Gel Purification Buffer, and a Melon Gel IgG Spin Purification Kit (Thermo Fisher) was used according to the manufacturer’s protocol. Purified IgG was used at a final concentration of 1:100 to generate immune complexes as described above.

### Neutrophil phagocytosis

Healthy polymorphonuclear neutrophils were obtained by treating healthy whole blood with ACK lysis buffer (Thermo Fisher) for 5 min at room temperature, followed by centrifugation and a PBS wash. Cells were resuspended in RPMI medium (10% FBS, 1% penicillin/streptomycin, Hepes, and L-glutamine), and 50,000 cells were incubated per well of immune complexes generated as described above in 200 μl for 1 h at 37°C. Cells were pelleted and stained for CD66b, CD14, and CD3 and analyzed with a BD LSRII flow cytometer to quantify MFI and percentage of CD3^−^CD14^−^CD66b^+^FITC^+^ cells. ADNP score was calculated by (FITC MFI) × (% FITC^+^)/10,000 for each well, and the average of duplicate wells was calculated. For FcγR blocking experiments, 1 × 10^6^ neutrophils were incubated with 10 μg of anti-human CD16 IgG (MEM154; Abcam), anti-human CD32 (IV3; BioXCell), or isotype IgG (purified mouse IgG1 or mouse IgG2b) for 1 h at 37°C, washed in PBS, and then used for ADNP.

### Microscopy

For NET analysis, 40,000 polymorphonuclear neutrophils were plated on poly-L-lysine–coverslips in a 24-well plate for 15 min at 37°C, and unbound cells were washed off with PBS. Each well was incubated with 500 μl of 0.1 μg immune complexes as described above for 2–3 h at 37°C. Cells were fixed with 4% paraformaldehyde for 15 min at room temperature, washed in PBS, and blocked overnight in 2 mM EDTA PBS containing 10% FBS, 1% BSA, and 0.05% Tween 20 at 4°C. Cells were sequentially stained with the following antibodies: mouse anti-neutrophil elastase (MABS461; Millipore) at 1:250, Cy3 anti-mouse IgG (Jackson ImmunoResearch) at 1:1,000, rabbit anti-histone H3 (Ab5103; Abcam) at 1:250, and Cy5 anti-rabbit IgG (Jackson ImmunoResearch) at 1:1,000. Cells were then stained with Hoechst 33342 (Thermo Fisher) at 1:100 for 10 min at room temperature, and ProLong Gold Antifade Mountant (Thermo Fisher) was added along with coverslips. A Leica TCS SP8 confocal microscope using the 40× immersion lens was used to obtain images. NETs were quantified by measuring the area of Hoechst staining (square millimeter) per cell in ImageJ. For each condition per donor plasma, four images were recorded, four cells were measured per image, and the average area per image was plotted.

### Antibody profiling analysis

Antibody subclass profiling analysis was performed as previously described (Brown et al., 2012). Briefly, recombinant K102, C1q, ssDNA, Ro-SSA, collagen, HA, or tetanus toxin was coupled to MagPlex beads (Luminex) via sulfo-N-hydroxysulfosuccinimide (NHS) coupling chemistry. Samples were diluted 1:1,000 (IgG1) or 1:100 (IgG2, IgG3, and IgG4) in 1× PBS + 0.1% BSA + 0.05% Tween20 (assay buffer) and incubated with antigen-coupled beads for 2 h at room temperature with shaking. Beads were washed, and different antibody subclasses (IgG1, IgG2, IgG3, or IgG4) were detected by incubating with 0.65 µg/ml of PE-labeled secondary antibodies (Southern Biotech) for 1 h at room temperature with shaking. Beads were washed and analyzed on a Flexmap 3D instrument (Luminex). The median fluorescent intensity of 30 beads/region was recorded. FcγR binding was measured as previously described (Brown et al., 2017). For glycan analysis, K102-conjugated Luminex beads were incubated overnight at 4°C with plasma diluted 1:10 in PBS. Beads were washed and incubated with biotinylated lectins from Vector Labs or biotinylated anti-human IgG for 30 min at room temperature with shaking. Beads were washed again, incubated with SA-PE for 10 min at room temperature with shaking, and washed before analysis on Bio-Plex instrument. MFI for each lectin was normalized by MFI for human IgG for each sample.

### Statistical analysis

Graph Pad Prism (v8.0) was used for all statistical analysis. Non-parametric Mann–Whitney *t* test was performed to calculate significance between groups. To compare more than two groups, we used one-way ANOVA Kruskal–Wallis test. Spearman r was calculated to determine significant correlation. Data are represented as means ± SEM. In all cases, *, P < 0.05; **, P < 0.01; ***, P < 0.001; ****, P < 0.0001; and ns, not significant.

### Online supplemental material

Fig. S1 shows ERV-K102, K115, K106, and K110 envelope SU sequence similarity and lack of orthologous sequences in other vertebrates. Fig. S2 shows that expression of ERV-K is higher in females than in males and correlates with anti-RNP titer. Fig. S3 depicts correlation between ERV-K expression and differentially expressed transcription factors and antiretroviral factors. Fig. S4 shows ERV-K102 cDNA sequence analysis and ADNP with different K102 protein preps and other antigens. Fig. S5 illustrates glycan modifications on anti-ERV-K102 IgG and NET analysis in neutrophils from SLE patients. Table S1 lists envelope-coding ERV-K loci in the ERVmap database.

## Supplementary Material

Table S1 lists envelope-coding ERV-K loci in the ERVmap database.
